# Effectiveness of dolutegravir‐based regimens as either first‐line or switch antiretroviral therapy: data from the Icona cohort

**DOI:** 10.1002/jia2.25227

**Published:** 2019-01-20

**Authors:** Annalisa Mondi, Alessandro Cozzi‐Lepri, Alessandro Tavelli, Stefano Rusconi, Francesca Vichi, Francesca Ceccherini‐Silberstein, Andrea Calcagno, Andrea De Luca, Franco Maggiolo, Giulia Marchetti, Andrea Antinori, Antonella d'Arminio Monforte, Icona Foundation Study Group, M Andreoni, A Castagna, F Castelli, R Cauda, G Di Perri, M Galli, R Iardino, G Ippolito, A azzarin, G Rezza, F von Schloesser, P Viale, F Ceccherini‐Silberstein, A Cozzi‐Lepri, E Girardi, S Lo Caputo, C Mussini, M Puoti, CF Perno, C Balotta, A Bandera, S Bonora, M Borderi, A Capetti, MR Capobianchi, S Cicalini, A Cingolani, P Cinque, A Di Biagio, N Gianotti, A Gori, G Guaraldi, G Lapadula, M Lichtner, G Madeddu, F Maggiolo, L Monno, S Nozza, E Quiros Roldan, R Rossotti, S Rusconi, MM Santoro, A aracino, L Sarmati, I Fanti, L Galli, P Lorenzini, A Rodano’, M Macchia, A Tavelli, F Carletti, S Carrara, A Di Caro, S Graziano, F Petrone, G Prota, S Quartu, S Truffa, Italy A Giacometti, A Costantini, V Barocci, G Angarano, C Fabrizio, C Suardi, V i, G Verucchi, F Castelnuovo, C Minardi, B Menzaghi, C Abeli, B Cacopardo, B Celesia, J Vecchiet, K Falasca, A Pan, S Lorenzotti, L Sighinolfi, D Segala, P Blanc, G Cassola, C Viscoli, A lessandrini, N Bobbio, G Mazzarello, I Pozzetto, P Bonfanti, C Molteni, A Chiodera, P Milini, G Nunnari, G Pellicanò, G Rizzardini, F Bai, MC Moioli, R Piolini, AL Ridolfo, S Salpietro, C Tincati, C Puzzolante, C Migliorino, V Sangiovanni, G Borgia, V Esposito, F Di Martino, I Gentile, L Maddaloni, AM Cattelan, S Marinello, A Cascio, C Colomba, F Baldelli, E Schiaroli, G Parruti, F Sozio, G Magnani, MA Ursitti, A Cristaudo, V Vullo, R Acinapura, G Baldin, M Capozzi, M Rivano Capparucia, G Iaiani, A atini, I Mastrorosa, MM Plazzi, S Savinelli, A Vergori, M Cecchetto, F Viviani, P Bagella, B Rossetti, R Fontana Del Vecchio, D Francisci, C Di Giuli, P Caramello, GC Orofino, M Sciandra, M Bassetti, A ondero, G Pellizzer, V Manfrin, G Starnini, A alungo

**Affiliations:** ^1^ HIV/AIDS Department National Institute for Infectious Diseases “Lazzaro Spallanzani” IRCCS Rome Italy; ^2^ Institute for Global Health University College London London UK; ^3^ Icona Foundation Milan Italy; ^4^ Infectious Diseases Unit ASST FBF‐Sacco, DIBIC “L. Sacco” University of Milan Milan Italy; ^5^ Unit of Infectious Diseases Santa Maria Annunziata Hospital Firenze Italy; ^6^ Department of Experimental Medicine and Surgery University of Rome Tor Vergata Rome Italy; ^7^ Unit of Infectious Diseases Department of Medical Sciences Amedeo di Savoia Hospital University of Torino Turin Italy; ^8^ Infectious Diseases Unit University of Siena Siena Italy; ^9^ Unit of Infectious Diseases ASST‐PG23 Bergamo Italy; ^10^ Clinic of Infectious and Tropical Diseases Department of Health Sciences ASST Santi Paolo e Carlo University of Milan Milan Italy

**Keywords:** antiretroviral therapy, dolutegravir, cohort study, discontinuation, toxicity, adverse events

## Abstract

**Introduction:**

Concerns about dolutegravir (DTG) tolerability in the real‐life setting have recently arisen. We aimed to estimate the risk of treatment discontinuation and virological failure of DTG‐based regimens from a large cohort of HIV‐infected individuals.

**Methods:**

We performed a multicentre, observational study including all antiretroviral therapy (ART)‐naïve and virologically suppressed treatment‐experienced (TE) patients from the Icona (Italian Cohort Naïve Antiretrovirals) cohort who started, for the first time, a DTG‐based regimen from January 2015 to December 2017. We estimated the cumulative risk of DTG discontinuation regardless of the reason and for toxicity, and of virological failure using Kaplan–Meier curves. We used Cox regression model to investigate predictors of DTG discontinuation.

**Results:**

About 1679 individuals (932 ART‐naïve, 747 TE) were included. The one‐ and two‐year probabilities (95% CI) of DTG discontinuation were 6.7% (4.9 to 8.4) and 11.5% (8.7 to 14.3) for ART‐naïve and 6.6% (4.6 to 8.6) and 7.6% (5.4 to 9.8) for TE subjects. In both ART‐naïve and TE patients, discontinuations of DTG were mainly driven by toxicity with an estimated risk (95% CI) of 4.0% (2.6 to 5.4) and 2.5% (1.3 to 3.6) by one year and 5.6% (3.8 to 7.5) and 4.0% (2.4 to 5.6) by two years respectively. Neuropsychiatric events were the main reason for stopping DTG in both ART‐naïve (2.1%) and TE (1.7%) patients. In ART‐naïve, a concomitant AIDS diagnosis predicted the risk of discontinuing DTG for any reason (adjusted relative hazard (aRH) = 3.38, *p* = 0.001), whereas starting DTG in combination with abacavir (ABC) was associated with a higher risk of discontinuing because of toxicity (aRH = 3.30, *p* = 0.009). TE patients starting a DTG‐based dual therapy compared to a triple therapy had a lower risk of discontinuation for any reason (adjusted hazard ratio (aHR) = 2.50, *p* = 0.037 for ABC‐based triple‐therapies, aHR = 3.56, *p* = 0.012 for tenofovir‐based) and for toxicity (aHR = 5.26, *p* = 0.030 for ABC‐based, aHR = 6.60, *p* = 0.024 for tenofovir‐based). The one‐ and two‐year probabilities (95% CI) of virological failure were 1.2% (0.3 to 2.0) and 4.6% (2.7 to 6.5) in the ART naïve group and 2.2% (1.0 to 3.3) and 2.9% (1.5 to 4.3) in the TE group.

**Conclusions:**

In this large cohort, DTG showed excellent efficacy and optimal tolerability both as first‐line and switching ART. The low risk of treatment‐limiting toxicities in ART‐naïve as well as in treated individuals reassures on the use of DTG in everyday clinical practice.

## 1 Introduction

Despite its recent introduction, dolutegravir (DTG) is now one of the most used antiretroviral drugs, thanks to its high efficacy combined with convenient dosing, the lack of pharmacokinetic boosting requirement and the high barrier to resistance. It is currently recommended both as first‐line therapy and as part of either switching strategies or salvage regimens in pretreated patients in high‐income countries 1. Since July 2018, it is also recommended as a first‐line treatment option for resource‐limited countries by World Health Organization (WHO) treatment guidelines 2.

The efficacy of DTG in antiretroviral therapy (ART)‐naïve population has been widely demonstrated in randomized clinical trials, showing non‐inferiority compared to raltegravir 3, and superiority compared to both non‐nucleoside reverse transcriptase inhibitors 4 and protease inhibitors‐based regimens 5, 6. In these latter studies, the superiority of DTG was mainly driven by a better tolerability of the drug compared to its comparators, with a lower rate of treatment discontinuations 4, 5, 6. This elevated performance has been also confirmed in the treatment‐experienced (TE) population both as salvage therapy in heavily pretreated subjects 7, 8, and simplification strategy in virologically suppressed patients 9. In the latter context, DTG has also been investigated as part of less drug regimens. While simplification to DTG monotherapy has recently demonstrated suboptimal efficacy compared to triple ART 10, recent randomized trials have demonstrated the non‐inferiority to standard ART of the two‐drug regimens, DTG plus rilpivirine in virologically suppressed subjects 11 and DTG plus lamivudine (3TC) in ART‐naïve patients 12. This latter combination has also showed promising results as a switching option in a non‐randomized trial and in observational studies 13, 14, 15, whereas a large randomized clinical trial exploring this strategy in now ongoing.

Despite the optimal safety profile demonstrated in clinical trials 3, 4, 5, 6, 7, 8, 9, 10, 11, 12, recent data from observational studies have questioned the tolerability of DTG, reporting a high incidence of discontinuations due to toxicity, mainly related to neuropsychiatric events 16, 17, 18. Although these findings were not uniformly confirmed 19, 20, 21, further studies are needed to accurately estimate the tolerability profile of DTG‐based regimens in the real‐life setting, even considered the forthcoming broad use of DTG both as part of dual regimens in ART‐naïve and ‐experienced patients as well as first‐line option in resource‐limited countries 2.

This study aimed to estimate the risk of DTG‐discontinuation and virological failure DTG‐based regimens and to explore the predictive factors of DTG interruptions in ART‐naïve and virologically suppressed TE patients, from a large cohort of HIV‐infected individuals.

## 2 Methods

### 2.1 Study population

This is a retrospective analysis of prospectively collected data from the Icona (Italan Cohort Naïve Antiretrovirals) Foundation cohort. Icona is an observational cohort, set up in 1997, including HIV‐1‐infected subjects, naïve from ART at the time of enrolment, seen for care in Italy. To date, the cohort consists of more than 16,000 patients prospectively followed in 51 centres. Demographic, viro‐immunological and clinical data together with information on antiretroviral regimens are collected and recorded using an electronic database. Details of the cohort have been described elsewhere 22.

Both ART‐naïve and TE virologically suppressed patients enrolled in the Icona cohort who initiated, for the first time, any DTG‐based regimen from January 2015 to December 2017, were included in the analysis. ART‐experienced patients were considered virologically suppressed if their most recent available viral load before the DTG initiation was below 50 copies/mL. The reasons for DTG discontinuation, as reported by the treating physicians, were recorded and classified into the following subgroups: simplification (defined as either the reduction of drugs in the regimen or the decrease in daily doses/pills), toxicity (defined as either side effects potentially due to the prescribed drug or demonstrated toxicity to the drug), failure (either virological, immunological or clinical), adherence issues and unknown/other causes (e.g. death, pregnancy, drug–drug interactions, enrolment in clinical trials, physician's choice not otherwise specified).

### 2.2 Objectives

The primary objective was to estimate the risk of DTG discontinuation regardless of the reason. Secondary objectives were to assess the risk of DTG interruption due to toxicity and, specifically, to neuropsychiatric adverse events and the risk of virological failure. Moreover, factors independently associated with the risk of DTG discontinuation for any reason and for toxicity were identified. Only discontinuations of DTG were considered, regardless of whether the remaining antiretroviral drugs used in the combination had been stopped or not. Virological failure was defined at the time of the first of two consecutive HIV viral loads above the threshold of 50 copies/mL. In ART‐naïve patients, virological events were counted starting from six months after ART initiation.

### 2.3 Statistical analyses

Standard survival analysis by the Kaplan–Meier method was used to estimate the cumulative probability of primary and secondary endpoints as defined above with their respective 95% confidence intervals. We used a marginal model approach to investigate the cumulative risk of DTG stop due to both any and neuropsychiatric toxicity. In this model, the follow‐up of patients who discontinued for a reason different from general or neuropsychiatric toxicity was truncated at the date of last clinical follow‐up. For the endpoints involving DTG discontinuation participants’ follow‐up accrued from the date of DTG starting until its discontinuation or the last available clinical visit. For the virological failure endpoint censoring was applied at the date of participants’ last available viral load measure. An intention‐to‐treat approach (ignoring treatment change) was used for the virological failure analysis.

Multivariable Cox regression models were used to identify factors independently associated with the risk of DTG discontinuation for any reason and for toxicity. The following factors were *a priori* included as covariates in the model: age, gender, mode of HIV transmission, nationality, AIDS diagnosis, hepatitis coinfection, calendar year of starting DTG, most recent CD4 count and HIV RNA (only for ART‐naïve patients) at DTG initiation, type of DTG‐based regimen and, limited to TE patients, previous virological failure, reasons for stopping previous regimen, duration of ART and of virological suppression prior to DTG initiation. In the ART‐naïve group, DTG‐based regimens were stratified according to the backbone. Conversely, in TE group, considering the more heterogeneous types of regimens started, DTG‐based therapies were stratified according both to the backbone and to the type of regimen (abacavir (ABC)‐ vs. tenofovir‐based standard triple therapies vs. dual therapies). Since the two treatment groups were heterogeneous for almost all the main baseline characteristics, all analyses were performed separately for ART‐naïve and TE patients.

All statistical analyses were performed using SAS (version 9.4, SAS Institute, Cary, NC, USA). All *p*‐values presented are two sided and a *p*‐value < 0.05 indicated conventional statistical significance.

### 2.4 Ethics statement

The Icona Foundation study was approved by the Ethics Committee (institutional review board) of each participating institution. All of the individuals enrolled provided a written informed consent at the time of the enrolment. All procedures of the study were performed in accordance with the 1964 Helsinki declaration and its later amendments.

## 3 Results

### 3.1 Baseline patients’ characteristics

A total of 1679 HIV‐positive patients, 932 ART‐naïve and 747 TE, who started DTG for the first time in any antiretroviral regimen, were included in the analysis. The main characteristics of the study population at the time of DTG initiation, overall and according to prior ART history, are shown in Table 1. Briefly, of the 1679 subjects, 20% were female, the median age was 44 years (interquartile range, IQR 35 to 52), the majority of patients contracted HIV infection through sexual intercourses (48% men having sex with men, 37% heterosexual), only few individuals had a prior AIDS diagnosis (14%) or were co‐infected with hepatitis viruses (10%). DTG was started in 1402 patients (84%) in a standard triple therapy regimen (63% ABC‐based; 37% tenofovir disoproxil fumarate (TDF)/tenofovir alafenamide fumarate (TAF)‐based), in 218 patients (13%) as part of a two‐drug therapy and in 59 (4%) patients in other regimens. As expected, the two populations significantly differed for most of the baseline characteristics. Particularly, ART‐naïve patients were more likely to be younger (*p* < 0.001), male (*p* = 0.002), non‐Italian (*p* < 0.001) and to have a higher CD4 cell count nadir (*p* < 0.001). Almost all ART‐naïve patients (95%) started a standard DTG‐based triple therapy (52% ABC‐based, 48% tenofovir‐based) whereas, in the TE group, 70% of patients started DTG within a standard triple ART, mainly ABC‐based (81%) and 27% started DTG as part of a dual therapy (*p* < 0.001 compared to ART‐naïve subjects).

**Table 1:** Main baseline characteristics of total population and according to the treatment group

	ART‐naive	Treatment‐experienced	*p*‐Value	Total
	N = 932	N = 747		N = 1679
Gender, n (%)			**0.002**	
Female	159 (17.1)	172 (23.0)		331 (19.7)
Age, years			**<0.001**	
Median (IQR)	40 (32 to 49)	48 (39 to 55)		44 (35 to 52)
Mode of HIV transmission, n (%)			**<0.001**	
MSM	487 (52.9)	313 (42.0)		800 (48.0)
Heterosexual	313 (33.6)	299 (40.0)		612 (36.5)
IDU	38 (4.1)	91 (12.2)		129 (7.7)
Other/unknown	83 (9.0)	42 (5.6)		125 (7.5)
Nationality, n (%)			**<0.001**	
Non‐Italian	193 (20.7)	67 (9.0)		260 (15.5)
AIDS diagnosis, n (%)			**<0.001**	
Yes	108 (11.6)	129 (17.3)		237 (14.1)
HBsAg, n (%)			**<0.001**	
Negative	683 (73.3)	659 (88.2)		1342 (79.9)
Positive	1 (0.1)	12 (1.6)		13 (0.8)
Not tested	248 (26.6)	76 (10.2)		324 (19.3)
HCVAb, n (%)			**<0.001**	
Negative	655 (70.3)	581 (77.8)		1236 (73.6)
Positive	36 (3.9)	116 (15.5)		152 (9.1)
Not tested	241 (25.9)	50 (6.7)		291 (17.3)
Calendar year of baseline, n (%)			**<0.001**	
2015	217 (23.3)	225 (30.1)		442 (26.3)
2016	398 (42.7)	358 (47.9)		756 (45.0)
2017	317 (34.0)	164 (22.0)		481 (28.7)
CD4 count nadir, cells/mmc			**<0.001**	
Median (IQR)	345 (129 to 540)	272 (149 to 374)		301 (139 to 458)
Baseline CD4 count, cells/mmc			**<0.001**	
Median (IQR)	350 (127 to 562)	651 (463 to 862)		500 (276 to 726)
HIV RNA, log10 copies/mL				
>100,000 copies/mL, n (%)	349 (42.3)	–	–	349 (22.2)
Type of DTG‐based regimen started, n (%)				
Standard triple therapy	**882 (94.6)**	**520 (69.6)**	**<0.001**	**1402 (83.5)**
ABC‐based	461 (52.3)	422 (81.2)		883 (63.0)
TDF‐based	372 (42.2)	94 (18.1)		466 (33.2)
TAF‐based	49 (5.6)	4 (0.8)		53 (3.8)
Dual therapy	**14 (1.5)**	**204 (27.3)**	**<0.001**	**218 (13.0)**
DTG + RPV	2 (14.3)	34 (16.7)		36 (16.5)
DTG + 3TC	3 (21.4)	122 (59.8)		125 (57.3)
DTG + DRV/b	8 (57.1)	33 (16.2)		41 (18.8)
Other dual regimens	1 (7.1)	15 (7.4)		16 (7.3)
Other	**36 (3.9)**	**23 (3.1)**	0.386	**59 (3.5)**
DTG dosage (%)			**0.024**	
QD	923 (99.0)	742 (99.3)		1665 (99.2)
BID	9 (1.0)	5 (0.7)		14 (0.8)
History of failure, n (%)			–	
Any failure	–	259 (34.6)	–	259 (15.4)
Failure to INSTI		17 (2.3)		17 (1.0)
Duration of VS ≤ 50 copies/mL, months			–	
Median (IQR)		43 (21 to 80)		
Follow‐up time, months			**<0.001**	
Median (IQR)	9 (3 to 17)	13 (6 to 20)		11 (5 to 18)

ART, antiretroviral therapy; IQR, interquartile range; MSM, men sex with men; IDU, intravenous drug user; Ag, antigen; Ab, antibodies; ABC, abacavir; TDF, tenofovir disoproxil fumarate; TAF, tenofovir alafenamide fumarate; DTG, dolutegravir; RPV, rilpivrine; 3TC, lamivudine; DRV/b, darunavir/ritonravir or darunavir/cobicistat; QD, quaque die; BID, bis in die; INSTI, integrase strand transfer inhibitor; VS, viral suppression. Bold values in the third column represent statistically significant p‐values. Bold values in the first and second columns represent the total number of patient per class of antiretroviral regimen and are the sum of the number reported below in the column.

### 3.2 DTG discontinuation for any reason

Overall, over a median follow‐up of 11 months (IQR 5 to 18), 121 subjects (7.2%) stopped DTG, of whom 71 were in the ART‐naïve group (7.6%) and 50 were in the TE group (6.7%). Reasons for DTG discontinuations were similar across the two groups. Specifically, toxicity was the most common cause for treatment discontinuation in both ART‐naïve and TE patients (55% and 54% of DTG discontinuations) followed by lack of efficacy (11% in ART‐naïve vs. 6% in TE), simplification (8% vs. 10%) and adherence issues (4% vs. 6%) (Table 2).

**Table 2:** Reasons for DTG discontinuation regardless of the reason and due to toxicity according to treatment group

	ART‐naïve	TE
Reasons for DTG discontinuation (n (% population))^a^		
Toxicity	39 (4.2)	27 (3.6)
Lack of efficacy	8 (0.9)	3 (0.4)
Simplification	6 (0.6)	5 (0.6)
Adherence issues	3 (0.3)	3 (0.4)
Other/unknown	15 (1.6)	12 (1.6)
AEs Leading to DTG discontinuation (n (% population))^a^		
Neuropsychiatric	20 (2.1)	13 (1.7)
Gastrointestinal	3 (0.3)	6 (0.8)
Allergic reactions	9 (1.0)	–
Hepatic	3 (0.3)	1 (0.1)
Osteoarticular	–	3 (0.4)
Renal	1 (0.1)	2 (0.3)
Other/unknown	3 (0.3)	2 (0.3)
Neuropsychiatric AEs leading to DTG discontinuation (n)^b^		
Insomnia	7	4
Depression	4	1
Anxiety and mood disorders	4	1
Paraesthesia	2	1
Dizziness	1	2
Headache	2	3
Suicidal ideation	2	0
Other neurological AEs^c^	5	1
Other psychiatric AEs^d^	2	0
Not specified	1	0

ART, antiretroviral therapy; TE, treatment‐experienced; DTG, dolutegravir; AEs, adverse events.

^a^For each patient, only one main reason for DTG discontinuation and one category of toxicity leading to DTG discontinuation is possible; ^b^more than one neuropsychiatric symptoms for each patient is possible; ^c^ART‐naïve: anosmia, photophobia, visual disturbances, cognitive‐motor slowing; TE: tinnitus; ^d^ART naïve patients: paranoid behaviour, hallucinations.

**Figure 1:**
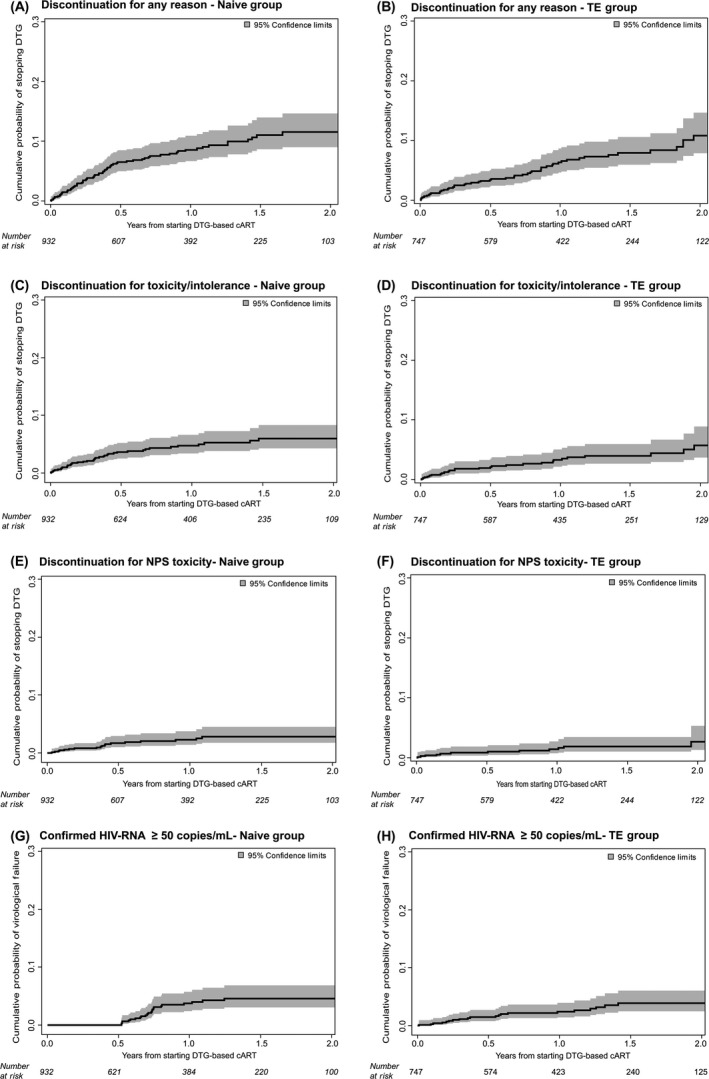
Kaplan–Meier curves estimating cumulative probability of dolutegravir (DTG)‐discontinuation regardless of the reason (A,B), for toxicity (C,D) and for neuropsychiatric adverse events (E,F) and the cumulative probability of virological failure (G,H) in antiretroviral therapy (ART)‐naïve and treatment‐experienced (TE) groups

In the ART‐naïve group, the one‐ and two‐year estimated probability of discontinuing DTG regardless of the reason were 6.7% (95% CI: 4.9 to 8.4) and 11.5% (95% CI: 8.7 to 14.3) respectively (Figure 1A). At multivariable Cox regression analysis, after adjusting for potential confounders listed in the Methods, patients diagnosed with an AIDS‐defining event were independently associated with a higher risk of stopping DTG for any reason (adjusted hazard ratio, aHR = 3.38, *p* = 0.001) (Table 3A). The AIDS‐defining events which occurred in 17 of 71 naïve patients who discontinued DTG were: systemic Cytomegalovirus infection without end‐organ disease (n = 7), *Pneumocystis jirovecii* pneumonia (n = 5), esophageal candidiasis (n = 4), tuberculosis (n = 3), Kaposi sarcoma (n = 3), cerebral toxoplasmosis (n = 2), non‐Hodgkin lymphoma (n = 2), cervical cancer (n = 1) and AIDS dementia complex (n = 1). More than one event for a patient could be reported. Of note, five cases of immunoreconstituition inflammatory syndrome were reported in our ART‐naïve population (0.5% of all ART naïve patients, 4.6% of ART‐naïve patients with a concurrent AIDS diagnosis). Among them, just one patient discontinued DTG.

**Table 3:** Predictors of DTG discontinuation for any reason and for toxicity by multivariable Cox regression models according to treatment group (A: ART‐naïve group and B: TE group)

(A) ART‐naïve group Variables	Discontinuation for any reason		Discontinuation for toxicity	
	aRH^a^ (95% CI)	*p*‐Value	aRH^a^ (95% CI)	*p*‐Value
Gender				
Female	1.46 (0.67 to 3.19)	0.340	1.48 (0.45 to 4.84)	0.515
Age, years				
Per 10 older	1.15 (0.94 to 1.40)	0.181	1.26 (0.88 to 1.79)	0.208
AIDS diagnosis				
Yes vs. no	3.38 (1.62 to 7.05)	**0.001**	2.82 (0.96 to 8.28)	0.060
Calendar year of baseline				
Per more recent year	1.26 (0.81 to 1.95)	0.313	1.37 (0.74 to 2.52)	0.318
Baseline CD4 count, cells/mm^c^				
Per 100 higher	0.98 (0.86 to 1.11)	0.730	0.96 (0.81 to 1.13)	0.601
HIV‐RNA, log10 copies/mL				
Per log higher	1.27 (0.87 to 1.84)	0.216	1.13 (0.69 to 1.87)	0.623
NRTI backbone				
TDF or TAF/FTC	1.00		1.00	
3TC/ABC	1.39 (0.79 to 2.46)	0.253	3.30 (1.34 to 8.11)	**0.009**

(B) TE group Variables	Discontinuation for any reason		Discontinuation for toxicity	
	aRH^b^ (95% CI)	*p*‐Value	aRH^b^ (95% CI)	*p*‐Value
Gender				
Female	1.11 (0.52 to 2.33)	0.799	1.78 (0.66 to 4.78)	0.255
Age, years				
Per 10 older	1.07 (0.81 to 1.41)	0.639	0.97 (0.66 to 1.42)	0.869
AIDS diagnosis				
Yes vs. no	1.32 (0.64 to 2.72)	0.450	1.35 (0.50 to 3.68)	0.552
Calendar year of baseline				
Per more recent year	1.06 (0.60 to 1.88)	0.839	0.89 (0.43 to 1.87)	0.767
DTG‐regimen^c^				
Dual	1.00		1.00	
Triple with ABC	2.50 (1.06 to 5.93)	**0.037**	5.26 (1.17 to 23.56)	**0.030**
Triple with TDF/TAF	3.56 (1.33 to 9.53)	**0.012**	6.60 (1.29 to 33.85)	**0.024**
Duration of VS				
Per six months longer	0.94 (0.88 to 0.99)	**0.028**	0.94 (0.83 to 1.07)	0.352

ART, antiretroviral therapy; TE, treatment‐experienced; aRH, adjusted relative hazard; CI, confidence intervals; NRTI, nucleoside reverse transcriptase inhibitor; FTC, emtricitabine; 3TC, lamivudine; DTG, dolutegravir; ABC, abacavir; TDF, tenofovir disoproxil fumarate; TAF, tenofovir alafenamide fumarate; VS, viral suppression. Bold values represent statistically significant *p*‐values.

^a^After adjusting mode of HIV transmission, nationality, hepatitis coinfection; ^b^after adjusting for mode of HIV transmission, nationality, hepatitis co‐infection, baseline CD4 cell count, reasons for stopping previous regimen, previous virological failure, duration of ART; ^c^other therapies group not shown because no events occurred.

In the ART‐experienced population, the Kaplan–Meier estimates of DTG interruption for any reason were 6.6% (95% CI: 4.6 to 8.6) and 7.6% (95% CI: 5.4 to 9.8) by one and two years respectively (Figure 1B). At multivariable analysis, TE patients starting DTG as part of a dual regimen compared to triple therapy, regardless of the backbone, had a lower risk of discontinuation for any cause (aHR 2.50 for ABC‐based triple therapies, *p* = 0.037; aHR 3.56 for tenofovir‐based triple therapies, *p* = 0.012) (Table 3B). A more durable virological suppression was also associated with a lower risk of interrupting DTG regardless of the reason (aHR per six months longer: 0.94, *p* = 0.028).

It is worth mentioning that five pregnancies occurred in our population over the observation time. All the five women were on DTG at the time of the conception. Three of them discontinued DTG within the first trimester of pregnancy, while two continued the drug throughout the entire pregnancy. No newborn safety issues have so far been reported.

### 3.3 Toxicity‐driven DTG discontinuation

Overall, DTG discontinuation was driven by toxicity in 66 patients (3.9% of total population, 55% of those who discontinued). All the adverse events were mild or moderate and none of them led to hospitalization or death. Neuropsychiatric adverse events, although rarely observed (2.0%), were the main cause of DTG discontinuation both for toxicity (33/66 (50%) of stops due to adverse events) and for any cause (33/121 (27%) of total stops). In our population, the most complained neuropsychiatric symptoms leading to DTG discontinuation were insomnia (n = 11), depression (n = 5), anxiety/mood disorders (n = 5) and headache (n = 5). Three patients in the ART‐naïve group reported suicidal ideation. A detailed description of the neuropsychiatric symptoms leading to DTG stop is shown in Table 2. A description of the toxicities leading to DTG discontinuation according to treatment group and gender is reported in Table S1.

Among ART‐naïve patients, 39 individuals (4.2%) stopped DTG for an adverse event. Neuropsychiatric events and allergic reactions were the most reported adverse events leading to DTG discontinuations (2.1% and 1.0% respectively) (Table 2). At survival analysis, the estimated one‐ and two‐year probabilities of discontinuing DTG because of adverse events were 4.0% (95% CI: 2.6 to 5.4) and 5.6% (95% CI: 3.8 to 7.5) respectively (Figure 1C). Restricting the analysis to neuropsychiatric toxicity, the estimated probabilities of interrupting DTG because of a neuropsychiatric event were 2.1% (95% CI: 1.0 to 3.1) by one year and 2.5% (95% CI: 1.3 to 3.8) by two years (Figure 1E). Having started DTG in combination with ABC compared to TDF or TAF was the only factor associated with a significantly higher risk of interrupting DTG for adverse events in previously untreated patients by multivariable analysis (aHR = 3.30, *p* = 0.009) (Table 3A). It is worth mentioning that none of the ART‐naïve patients who discontinued DTG for toxicity had changed the initial backbone before the event. In the TE group, 27 DTG discontinuations (3.6% of TE patients) were motivated by adverse events, mostly of a neuropsychiatric and gastrointestinal nature (1.7% and 0.8% respectively) (Table 2). At survival analysis, the one‐year estimated probability of interrupting DTG because of both any and neuropsychiatric adverse events in pretreated patients were 2.5% (95% CI: 1.3 to 3.6) and 1.2% (95% CI: 0.4 to 2.0) respectively. The two‐year estimated probabilities of discontinuation were 4.0% (95% CI: 2.4 to 5.6) for any adverse event and 1.7% (95% CI: 0.6 to 2.7) for neuropsychiatric toxicity (Figure 1D,F). At multivariable analysis, starting DTG as a part of a dual regimen compared to a standard triple therapy confirmed its association with a lower probability of interrupting DTG due to toxicity (aHR 5.26 for ABC‐based triple therapies, *p* = 0.030; aHR 6.60 for tenofovir‐based triple therapies, *p* = 0.024) (Table 3B). The significantly lower risk of DTG discontinuation for both any reason and for toxicity with dual regimens over triple therapies was also confirmed after adjusting the results for patients’ comorbidities (cardiovascular risk class, renal function, cholesterol levels) (data not shown).

### 3.4 Virological failure

Overall, during the observation time, virological failure occurred in 45 patients (2.7%) of whom 24 were ART‐naïve (2.6%) and 21 were TE (2.8%) patients. The Kaplan–Meier estimated probabilities of experiencing virological failure at one and two years of treatment were 1.2% (95% CI: 0.3 to 2.0) and 4.6% (95% CI: 2.7 to 6.5) in the ART naïve group and 2.2% (95% CI: 1.0 to 3.3) and 2.9% (95% CI: 1.5 to 4.3) in the TE group respectively (Figure 1G,H). Particularly, in the subgroup of 122 TE patients who switched to DTG plus 3TC dual regimen, only one patient (0.8%) experienced virological failure over the entire follow‐up. The genotype resistance test after virological failure, performed in 10 patients (four ART‐naïve and six TE), did not show the emergence of new major drug resistance mutations.

## 4 Discussion

In this large observational study, including 1679 HIV‐infected individuals (of whom 932 ART‐naïve) starting a DTG‐based therapy for the first time showed optimal efficacy and high tolerability with an overall low rates of virological failures (2.7%) and treatment discontinuations (7.2%). Consistently with data from postregistration studies 16, 17, 18, 19, 21 in our population, toxicity, mainly related to neuropsychiatric events, was the leading reason for stopping DTG. Particularly, in line with previous findings 17, 23, insomnia along with depression and mood disorders were the most frequently complained neuropsychiatric symptoms leading to DTG stop.

Nonetheless, the rates of DTG discontinuation due to adverse events and, specifically, to neuropsychiatric events observed in this study (3.9% and 2.0%, respectively) were considerably lower compared to several recent observational studies showing rates of DTG interruptions ranging from 7.6% to 13.7% 16, 17, 18, with the highest occurrence reported in the Dutch cohort (13.7%, 71% due to neuropsychiatric toxicity) 16. In the large cohort examined by Hoffman et al., although the rates of adverse events leading to treatment stop were similar between DTG and elvitegravir (7.6% at 12 months), the risk of discontinuing treatment for neuropsychiatric events was consistently higher for the former (5.6% vs. 0.7% at 12 months) 17. However, this increased risk of DTG discontinuation due to toxicity was not uniformly confirmed in real‐life settings. In fact, in line with our findings, several observational studies reported lower rates of discontinuation of DTG‐based regimens because of toxicity 19, 20, 21, with a tolerability profile comparable to the other integrase strand transfer inhibitors 19, 21. In particular, the Swiss HIV cohort study, reported rates of DTG discontinuation within the first year, due to both unspecified and neuropsychiatric toxicity, comparable to our results (3.9% and 1.7% respectively) 21. Currently, to the best of our knowledge, the rates of DTG discontinuation regardless of the reason and related to toxicity in our study are among the lowest reported in a real‐life setting. Of note, our results are comparable to those reported in randomized and non‐randomized clinical trials (1% to 12% discontinuations for any reason and 0% to 4% discontinuations due to adverse events after 12 months) which are, however, likely to include selected populations at lower risk of adverse events, therefore limiting the generalizability of the findings 3, 4, 5, 6, 7, 8, 9, 10, 11, 12 ^.^ The heterogeneous composition of study populations might partially explain the discrepant discontinuation rates between our study and previous observational reports. In particular, our results are based, as for the Swiss cohort 21, on a nation‐wide HIV cohort including patients from multiple different centres and, thus, are probably less influenced by patients’ selection or channelling biases than specifically designed observational prospective or retrospective databases.

Noteworthy, to date, our cohort includes one of the largest ART‐naïve population treated with DTG considering clinical trials as well as postmarketing studies. The low risk of toxicity limiting DTG treatment (5.6% by two years) and the negligible rates of neuropsychiatric events leading to DTG discontinuation (2.1%) observed in our ART‐naïve population are in line with preliminary pharmacovigilance data reported from a large Brazilian ART‐naïve cohort showing extremely low rates of DTG‐related adverse events (2.7%), mostly related to neuropsychiatric and gastrointestinal toxicities 24. Despite the differences in healthcare setting and HIV epidemiology, our data might add reassuring information in the light of the forthcoming broad use of DTG‐based first‐line therapies in middle‐ and low‐income countries after the recent update of WHO guidelines recommendations 2. Although DTG rollout in resource‐limited countries is predicted to be not only effective but also cost‐effective, particularly in view of the increasing prevalence of pretreatment NNRTI resistance 25, several questions on DTG safety remain to be addressed, particularly in special sub‐populations as severely immunocompromised patients and pregnant women 26, 27. In this latter group, surveillance data from Botswana cohort have recently reported potential risk of neural tube defects in infants born to women who were taking DTG at the time of conception 28, leading to the release of a safety alert on DTG use in pregnancy 29_._ Although no birth defects were reported in women exposed to DTG at the time of conception in our cohort, the few pregnancies that occurred, the low number of women of childbearing age and the deficiency on pregnancy outcomes, do not allow this study to address any concerns on DTG use during pregnancy.

Concerning predictive factors of DTG interruption, in our ART‐naïve population, a concurrent AIDS diagnosis predicted a higher risk of discontinuing DTG regardless of the reason. Toxicity issues, potential pharmacological interactions along with compromised patients’ clinical condition could partially explain this finding. Ongoing clinical trials with less restrictive exclusion criteria will probably contribute to better clarify this association, which assume a remarkable importance in the light of the future extensive use of DTG in resource‐limited countries where HIV diagnosis and ART initiation generally occurs at a more advanced stage of the disease.

Consistently with previous findings 16, 17, in our cohort, ART‐naïve patients starting DTG in combination with ABC were at higher risk of experiencing adverse events leading to treatment discontinuation compared to TAF or TDF. Pharmacokinetic‐driven drug–drug interaction, initially postulated to explain this result 16, has been contradicted by further evidence 30. This finding, to date not confirmed in previously randomized clinical trials in which DTG and ABC were co‐administrated 3, 6, should be taken with caution because potential bias due to selection or due to an enhanced awareness of treating physicians on DTG tolerability cannot be ruled out.

In the TE group, patients starting a DTG‐based dual therapy showed a lower risk of drug interruption for any reason and for toxicity as compared to the subjects on a triple therapy, even after considering patients’ baseline comorbidities and history of previous failure. However, channelling bias cannot be completely ruled out. Indeed, heavily pretreated patients with fewer therapeutic options left or more adherent patients could be preferentially selected by physicians to start a less‐drug regimen than a triple therapy. Although GEMINI trials did not show any differences in terms of discontinuations for toxicity between DTG plus 3TC and DTG‐based triple therapies in ART‐naïve patients 12, data from controlled clinical trials comparing DTG‐based dual and triple ART in TE patients are awaited in order to verify this result. If the low risk of discontinuation of DTG‐based dual versus triple regimens in pretreated patients is confirmed, the choice of DTG‐based dual regimens as switching strategy will become more attractive, adding the advantage of the good tolerability to the already demonstrated high potency, low potential for drug interactions and low cost.

This study is not without limitations, including the observational nature of the study, which is prone to bias due to unmeasured confounders, the short follow‐up duration with a median less than one year, the possible patient selection biases, the incomplete information available about concomitant medications and the potential inaccurate reporting of reasons for DTG discontinuation. Nonetheless, this study has it strengths in the description of the real‐life setting and the large sample size, especially for the ART‐naïve patients.

## 5 Conclusions

In conclusion, we provided further evidence that DTG has an excellent virological efficacy in both ART‐naïve and TE patients. Furthermore, our data suggest a high tolerability of DTG‐based regimens as first‐line therapy in ART‐naïve patients as well as switching strategy in TE virologically suppressed patients, thus reassuring on the use of DTG in clinical practice. Although adverse events, especially neuropsychiatric symptoms, represented the main reason for stopping DTG, their frequency was relatively low. Severely immunocompromised ART‐naïve patients appeared to be at higher risk of DTG discontinuation and, thus, warrant a closer monitoring. While awaiting for the results of randomized trials comparing dual versus triple DTG‐based therapies in TE aviremic patients, our data offer an insight into this question.

## Competing interests

AM, ACL, AT, FM and FV have nothing to disclose. SR outside the submitted work reports grants, personal fees and non‐financial support from ViiV Healthcare, Gilead Sciences and Janssen‐Cilag, personal fees and non‐financial support from Bristol Myers Squibb and personal fees from Merck Sharp and Dohme. FCS outside the submitted work reports personal fees from Gilead Sciences, Bristol Meyers Squibb, Abbvie, Roche Diagnostic, Janssen‐Cilag, Abbott Molecular and ViiV Healthcare, grants and personal fees from Merck Sharp and Dohme and grants from Italian Ministry of Instruction University and Research. AC outside the submitted work reports grants and personal fees from ViiV Healthcare and personal fees from Merck Sharp and Dohme and Janssen‐Cilag. ADL outside the submitted work reports grants and personal fees from ViiV Healthcare, Merck Sharp and Dohme and Gilead Sciences and personal fees from Janssen‐Cilag and Abbvie. GM outside the submitted work reports personal fees from Gilead Sciences and ViiV Healthcare. AA outside the submitted work reports grants, personal fees and non‐financial support from Gilead Sciences, ViiV Healthcare and Bristol Myers Squibb, grants and personal fees from Janssen‐Cilag, personal fees from Merck Sharp and Dohme and personal fees and non‐financial support from Abbvie. AdAM outside the submitted work reports grants and personal fees from Gilead Sciences, ViiV Healthcare and Merck Sharp and Dohme and personal fees from Janssen and Bristol Myers Squibb.

## Authors’ contributions

AM designed the study, wrote the first draft of the manuscript and referred to appropriate literature. AdAM and AA coordinated the Icona Foundation, conceived and supervised the study and finalized the draft of the manuscript. ACL contributed to the design of the study, was the main responsible person for data analysis and contributed to the article drafting. AT was the main responsible person for the data collection and contributed to data interpretation and article drafting. SR, FM and ADL contributed to data interpretation and article drafting. FV, FCS, AC and GM reviewed the manuscript. All authors agreed with final article submission.

## Supporting information


**Table S1.** Adverse events leading to dolutegravir discontinuation according to treatment group and gender

## Appendix 1

### Icona Foundation Study Group

BOARD OF DIRECTORS: A d'Arminio Monforte (President), A Antinori (Vice‐President), M Andreoni, A Castagna, F Castelli, R Cauda, G Di Perri, M Galli, R Iardino, G Ippolito, A Lazzarin, GC Marchetti, G Rezza, F von Schloesser, P Viale.

SCIENTIFIC SECRETARY: A d'Arminio Monforte, A Antinori, A Castagna, F Ceccherini‐Silberstein, A Cozzi‐Lepri, E Girardi, S Lo Caputo, C Mussini, M Puoti, CF Perno.

STEERING COMMITTEE: A Antinori, C Balotta, A Bandera, S Bonora, M Borderi, A Calcagno, A Capetti, MR Capobianchi, A Castagna, F Ceccherini‐Silberstein, S Cicalini, A Cingolani, P Cinque, A Cozzi‐Lepri, A d'Arminio Monforte, A De Luca, A Di Biagio, E Girardi, N Gianotti, A Gori, G Guaraldi, G Lapadula, M Lichtner, S Lo Caputo, G Madeddu, F Maggiolo, G Marchetti, L Monno, C Mussini, S Nozza, CF Perno, C Pinnetti, M Puoti, E Quiros Roldan, R Rossotti, S Rusconi, MM Santoro, A Saracino, L Sarmati.

STATISTICAL AND MONITORING TEAM: A Cozzi‐Lepri, I Fanti, L Galli, P Lorenzini, A Rodano’, M Macchia, A Tavelli.

BIOLOGICAL BANK INMI: F Carletti, S Carrara, A Di Caro, S Graziano, F Petrone, G Prota, S Quartu, S Truffa.

PARTICIPATING PHYSICIANS AND CENTRES: Italy A Giacometti, A Costantini, V Barocci (Ancona); G Angarano, L Monno, C Fabrizio (Bari); F Maggiolo, C Suardi (Bergamo); P Viale, V Donati, G Verucchi (Bologna); F Castelnuovo, C Minardi, E Quiros Roldan (Brescia); B Menzaghi, C Abeli (Busto Arsizio); B Cacopardo, B Celesia (Catania); J Vecchiet, K Falasca (Chieti); A Pan, S Lorenzotti (Cremona); L Sighinolfi, D Segala (Ferrara); P Blanc, F Vichi (Firenze); G Cassola, C Viscoli, A Alessandrini, N Bobbio, G Mazzarello (Genova); M Lichtner, I Pozzetto (Latina); P Bonfanti, C Molteni (Lecco); A Chiodera, P Milini (Macerata); G Nunnari, G Pellicanò (Messina); A d'Arminio Monforte, M Galli, A Lazzarin, G Rizzardini, M Puoti, A Castagna, F Bai, MC Moioli, R Piolini, AL Ridolfo, S Salpietro, C Tincati (Milano); C Mussini, C Puzzolante (Modena); C Migliorino, G Lapadula (Monza); V Sangiovanni, G Borgia, V Esposito, F Di Martino, I Gentile, L Maddaloni (Napoli); AM Cattelan, S Marinello (Padova); A Cascio, C Colomba (Palermo); F Baldelli, E Schiaroli (Perugia); G Parruti, F Sozio (Pescara); G Magnani, MA Ursitti (Reggio Emilia); M Andreoni, A Antinori, R Cauda, A Cristaudo, V Vullo, R Acinapura, G Baldin, M Capozzi, S Cicalini, A Cingolani, M Rivano Capparucia, G Iaiani, A Latini, I Mastrorosa, MM Plazzi, S Savinelli, A Vergori (Roma); M Cecchetto, F Viviani (Rovigo); G Madeddu, P Bagella (Sassari); A De Luca, B Rossetti (Siena); A Franco, R Fontana Del Vecchio (Siracusa); D Francisci, C Di Giuli (Terni); P Caramello, G Di Perri, S Bonora, GC Orofino, M Sciandra (Torino); M Bassetti, A Londero (Udine); G Pellizzer, V Manfrin (Vicenza); G Starnini, A Ialungo (Viterbo).
