# Supplementary material for: Effectiveness of dolutegravir‐based regimens as either first‐line or switch antiretroviral therapy: data from the Icona cohort
Source: J Int AIDS Soc. 2019 Jan 20;22(1):e25227. doi: 10.1002/jia2.25227 (PMC6340053; doi:10.1002/jia2.25227)
Supplement: Supplementary file 1 — Table S1. Adverse events leading to dolutegravir discontinuation according to treatment group and gender [file JIA2-22-e25227-s001.docx]

**Supplementary table 1: Adverse events leading to dolutegravir discontinuation according to treatment group and gender**

|  | **AEs LEADING TO DTG DISCONTINUATION**  [n (% population)] # | | | | | | |
| --- | --- | --- | --- | --- | --- | --- | --- |
|  | **ART NAÏVE** | | **TE** | | **TOTAL** | |  |
|  | **Female**  **N=8 (5.0%)** | **Male**  **N=31 (4.0%)** | **Female**  **N=9 (5.2%)** | **Male**  **N=18 (3.1%)** | **Female**  **N=17 (5.1%)** | **Male**  **N=49 (3.6%)** |  |
| **TOXICITY** |  |  |  |  |  |  |  |
| - **Neuropsychiatric** | 2 (1.3%) | 18 (2.3%) | 3 (1.7%) | 10 (1.7%) | 5 (1.5%) | 28 (2.1%) |  |
| - **Gastrointestinal** | 0 (0.0%) | 3 (0.4%) | 6 (3.5%) | 0 (0.0%) | 6 (1.8%) | 3 (0.2%) |  |
| - **Allergic reactions** | 3 (1.9%) | 6 (0.8%) | 0 (0.0%) | 0 (0.0%) | 3 (0.9%) | 6 (0.4%) |  |
| - **Hepatic** | 1 (0.6%) | 2 (0.3%) | 0 (0.0%) | 1 (0.2%) | 1 (0.3%) | 3 (0.2%) |  |
| - **Osteoarticular** | 0 (0.0%) | 0 (0.0%) | 0 (0.0%) | 3 (0.5%) | 0 (0.0%) | 3 (0.2%) |  |
| - **Renal** | 0 (0.0%) | 1 (0.1%) | 0 (0.0%) | 2 (0.3%) | 0 (0.0%) | 3 (0.2%) |  |
| - **Other/Unknown** | 2 (1.3%) | 1 (0.1%) | 0 (0.0%) | 2 (0.3%) | 2 (0.6%) | 3 (0.2%) |  |
| (notes: ART= antiretroviral therapy; AEs= adverse events; DTG=dolutegravir; TE= treatment-experienced). | | | | | | | |
| # For each patient, only one category of toxicity leading to DTG discontinuation is possible. | | | | | | | |
